# COVID-19 vaccine acceptance and perceptions among dental teaching staff of a governmental university in Egypt

**DOI:** 10.1186/s42506-022-00104-6

**Published:** 2022-04-21

**Authors:** Mariam Sharaf, Omar Taqa, Haneen Mousa, Amira Badran

**Affiliations:** Dental Public Health & Pediatric Dentistry Department, Faculty of Dentistry, Ain Shams University, Cairo, Egypt

**Keywords:** COVID-19, Vaccine acceptance, Healthcare workers, Dentists, Teaching staff

## Abstract

**Background:**

Vaccine acceptance among healthcare workers (HCWs) is an important determinant of its acceptance among the general population. Dentists are an essential group of HCWs who are at an increased risk of COVID-19 infection. This study aimed to assess vaccine acceptance and its determinants among a group of dental teaching staff in Egypt.

**Methods:**

An Internet-based cross-sectional study was conducted where the dental teaching staff of a governmental university in Egypt were targeted using total population sampling. Data was collected on socio-demographics, attitudes towards COVID-19, risk perception, general attitudes towards vaccination, vaccine acceptance, and concerns about COVID-19 vaccines, along with barriers and motivators to vaccination. Multivariate regression was done to determine factors significantly associated with unwillingness to receive COVID-19 vaccine.

**Results:**

A total of 171 dental faculty members participated in the study. At the time of data collection (August 2021–October 2021), 45.6% of the dental teaching staff were willing to receive the vaccine, while 46.7% were against vaccination, and 7.6% were vaccine hesitant. Female gender, not having a private practice, not intending to travel internationally, having anyone sick in the immediate social circle, and being more anxious about COVID-19 were significantly associated with unwillingness to receive the COVID-19 vaccine.

**Conclusion:**

At the time of conducting this study (August 2021–October 2021), less than half of the participating dental teaching staff in the studied Egyptian university were willing to receive the COVID-19 vaccine. Findings of the current study can guide Egyptian health authorities to adopt strategies that correct misconceptions among HCWs, educate them and build their trust in the efficacy and safety of COVID-19 vaccines, which can ultimately increase its acceptance in the general population.

## Introduction

In March 2020, the World Health Organization (WHO) declared COVID-19 a global pandemic. Spreading mainly through human-to-human transmission, COVID-19 has infected around 269 million people worldwide at the time of this writing, and was responsible for the deaths of approximately 5.3 million people globally [1]. The rapid spread of the virus has elicited a global response from all countries to mitigate this crisis through strict quarantine and social distancing [2]. Despite such efforts, COVID-19 has continued to spread all over the globe, thus more effective methods, such as vaccines, had to be sought out in order to combat such a devastating virus. COVID-19 vaccine manufacturing varies greatly in terms of the underlying process for the vaccine production, ranging from recombinant, live attenuated, and inactivated vaccines [3]. Despite this, unwillingness to receive the vaccine remains a major threat that could undermine these efforts [4].

Healthcare workers (HCWs) are on the frontline against COVID-19, this puts them in a high-risk category for COVID-19 exposure and infection [5], and hence makes them a priority group for receiving the vaccine. Dental HCWs, in particular, are at a higher risk of exposure to COVID-19 due to the nature of their work, which requires them to be in direct contact with body fluids [6].

The success of any vaccination program relies greatly on HCWs [7]. This is attributed to the fact that their opinions influence acceptance, adherence, and hesitancy about vaccines among the general population [8]. Therefore, it is of extreme importance to estimate the levels of acceptance among HCWs [9]. Previous literature has indicated different levels of acceptance of COVID-19 vaccine among HCWs [10–14], which might be influenced by various factors, such as, country of residence, gender, age, income, perceived benefits and risks, and safety concerns [7, 15].

In Egypt, there have been around 365,831 confirmed cases and 20,877 deaths, by December 2021 [16]. Despite that, previous studies have shown low acceptance of COVID-19 vaccine among Egyptian HCWs [2, 11]. Undoubtedly, the attitudes of teaching staff members, in particular, have a major influence on the perceptions of their students, other dental HCWs, and hence, the public. This study aimed to explore the levels of COVID-19 vaccine acceptance and its determinants among a group of dental teaching staff (DTS), as well as their perceptions towards the vaccine.

## Methods

### Study design and population

A cross sectional study was conducted between August 2021 and October 2021 at the Faculty of Dentistry, Ain Shams University (FD-ASU), a governmental university located in Cairo, Egypt. To be eligible to participate in the study; participants of both genders, must be staff members or assisting staff at different specialties, who are currently residing in Egypt and working at FD-ASU and provided a consent to participate in the study. Participants who refused to consent for participation were excluded from the study.

### Sample size

Total population sampling, a type of purposive sampling, was used to recruit the entire working staff at FD-ASU, where a total of 273 participants were targeted, among which 171 participants completed the questionnaire. This sampling method was chosen due to the foreknown number of working staff members, which is relatively small, in addition to the accessibility of the whole study population, which would reduce selection bias.

### Study procedures

#### Recruitment

Participants were targeted via WhatsApp online platform by sending a brief message explaining the objectives of the study, along with a Google forms link for the Internet-based survey and the electronic consent. Online method of survey dissemination was chosen to adhere to the guidelines of physical distancing provided by Centers for Disease Control and Prevention (CDC) to mitigate the pandemic.

#### Study instrument

A structured Internet-based, self-administered questionnaire was developed in the English language after reviewing studies with similar objectives [5, 9, 17, 18] and was reviewed by three public health experts to ensure face and content validity. Afterwards, a pilot study was conducted to pre-test the length of the questionnaire and identify any language or structure issues. According to the feedback received, some questions were omitted, and others were clarified, and the final version of the questionnaire was then confirmed.

The final version of the questionnaire consisted of a total of 47 close-ended questions. Data were collected from participants on their age, gender, title and area of specialty, involvement in direct patient care, future intentions for international travel, and medical condition. Moreover, participants were asked about their sources of COVID-19 information, and whether the participants themselves or someone in their close network has previously contracted COVID-19.

Perceptions of participants towards the pandemic were explored by inquiring about measures taken by them against COVID-19 infection. Participants were also asked to rate their degree of anxiety about COVID-19, as well as their adherence to quarantine on a scale of “extremely,” “somewhat,” or “not at all.” Perceived risks of COVID-19 pandemic were also assessed using a 5-point Likert scale (from strongly agree to strongly disagree).

General perceptions towards vaccinations were inquired about by investigating the history of previous vaccine refusal/delay, where participants were required to respond with “yes,” “no,” or “I don’t remember.” Participants’ beliefs about safety and effectiveness of vaccines were also explored using a 5-point Likert scale (from strongly agree to strongly disagree).

Vaccine acceptance was assessed using one question “Are you willing to take COVID-19 vaccine?”. This question was used to indicate the overall willingness/acceptance of participants to receive the vaccine, which is the outcome of interest of this study. Responding with “yes” indicated vaccine acceptance, whereas “no” indicated vaccine refusal, and “not sure” indicated vaccine hesitancy.

Finally, perceived barriers and motivators to COVID-19 vaccine acceptance were explored using 14 questions on a 5-point Likert scale (from strongly agree to strongly disagree). These included questions about concerns regarding COVID-19 vaccine’s safety, efficacy, long-term side effects, teratogenicity, allergic reactions, and the short period of clinical trials.

#### Incentives

Each participant received an appreciation message for participating in the study, as well as a copy of the most recently published guidelines by WHO and CDC regarding COVID-19 infection prevention and vaccination.

### Statistical analysis

Data was analyzed using IBM SPSS® version 26. Categorical variables were presented in frequencies and percentages and were analyzed using Fisher’s exact test. Binary logistic regression was used to calculate the odds ratio (OR) and 95% confidence intervals (95% CI). Willingness to receive the vaccine was dichotomized such that participants who answered “not sure” or “no” were considered unwilling to receive the vaccine while those who answered “yes” were considered willing. Factors that were statistically significant in the bivariate analysis were included in the multivariate regression model. The level of statistical significance was set at *p* < 0.05.

## Results

**Fig. 1:**
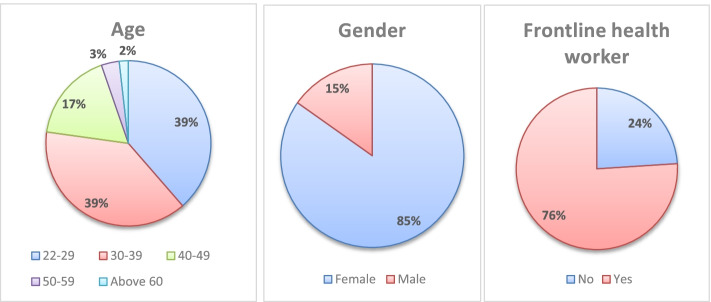
Sociodemographic data of participating dental teaching staff of the Faculty of Dentistry, Ain Shams University (*N* = 171)

**Fig. 2:**
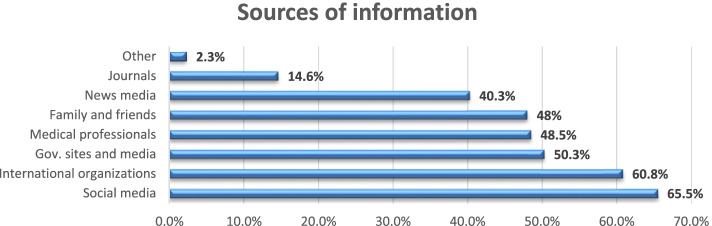
Sources of information of participating dental teaching staff of the Faculty of Dentistry, Ain Shams University (*N* = 171)

**Fig. 3:**
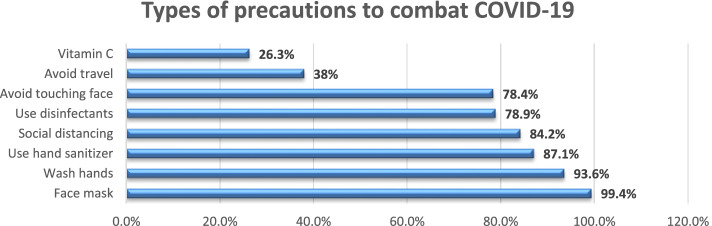
Types of precautions taken to combat COVID-19 by the participating dental teaching staff of the Faculty of Dentistry, Ain Shams University, Egypt (*N* = 171)

In total, there were 171 responses out of the 273 registered faculty members to whom the survey was originally sent (62.6% response rate). About two thirds of the sample were aged under 40 years. The majority of the respondents were females (84.8%), medically free (87.7%), and considered themselves to be frontline HCWs (76%) (involved in providing direct patient care) (Fig. 1). Social media was the most frequently used source of information (65.5%), followed by information shared by international organizations (60.8%) (Fig. 2). Almost all participants reported wearing facemasks as a precaution (99.4%), and (93.6%) reported washing hands (Fig. 3).

Only 19.3% of participants were sure they have not contracted COVID-19 so far, while only 21.6% were sure that no one in their immediate circle was sick at the time with COVID-19. Additionally, a small percentage of participants reported not being anxious about COVID-19 at all (6.4%), or not following the guidelines of quarantine at all (9.4%) (Table 1).

**Table 1:** Characteristics of the vaccine accepting, vaccine refusing, and vaccine hesitant dental teaching staff of the Faculty of Dentistry, Ain Shams University, Egypt (*N* = 171)

	Are you willing to take COVID-19 vaccine?			*P* value	Total frequency (percent)
	No (*N* = 80)	Not sure (*N* = 13)	Yes (*N* = 78)		
1-Age					
22–29	37(56.1%)	3 (4.5%)	26 (39.4%)	0.27	66 (37.6)
30–39	28 (42.4%)	5 (7.6%)	33 (50.0%)		66 (37.6)
40–49	13 (43.3%)	3 (10.0%)	14 (46.7%)		30 (17.5)
50–59	1 (16.7%)	2 (33.3%)	3 (50.0%)		6 (3.5)
Above 60	1 (33.3%)	0 (0.0%)	2 (66.7%)		3 (1.8)
2-Gender					
Female	74 (51.0%)	12 (8.3%)	59 (40.7%)	0.01*	145 (84.8)
Male	6 (23.1%)	1 (3.8%)	19 (73.1%)		26 (15.2)
3-Are you a frontline health worker?					
No	16 (39.0%)	6 (14.6%)	19 (46.3%)	0.13	41 (24)
Yes	64 (49.2%)	7 (5.4%)	59 (45.4%)		130 (76)
4-Current dentistry practice					
Faculty of Dentistry	48 (52.7%)	9 (9.9%)	34 (37.4%)	0.06	91 (53.2)
Faculty of Dentistry and Private clinic	32 (40.0%)	4 (5.0%)	44 (55.0%)		80 (46.8)
5- Intention to travel internationally					
No	73 (51)	12 (8.4)	58 (40.6)	0.01*	143 (83.6)
Yes	7 (25)	1 (3.6)	20 (71.4)		28 (16.4)
6- Existing chronic illness?					
No	71 (47.3%)	10 (6.7%)	69 (46.0%)	0.46	150 (87.7)
Yes	9 (42.9%)	3 (14.3%)	9 (42.9%)		21 (12.3)
7- Individual contracted COVID-19					
No	41 (47.7)	6 (7)	39 (45.3)	0.97	86 (50.3)
Yes	39 (45.9)	7 (8.2)	39 (45.9)		85(49.7)
8- Immediate social network contracted COVID-19					
No	15 (31.9)	2 (4.3)	30 (63.8)	0.01*	47 (27.5)
Yes	65 (52.4)	11 (8.9)	48 (38.7)		124 (72.5)
9-Rate your degree of adherence to quarantine					
Extremely	13 (39.4%)	4 (12.1%)	16 (48.5%)	0.36	33 (19.3)
Somewhat	62 (50.8%)	8 (6.6%)	52 (42.6%)		122 (71.3)
Not at all	5 (31.3%)	1 (6.3%)	10 (62.5%)		16 (9.4)
10-Rate your degree of anxiety about COVID-19					
Extremely	24 (55.8%)	4 (9.3%)	15 (34.9%)	< 0.01*	43 (25.1)
Somewhat	56 (47.9%)	7 (6.0%)	54 (46.2%)		117 (68.4)
Not at all	0 (0.0%)	2 (18.2%)	9 (81.8%)		11(6.4)

*Statistically significant differences. All data was analyzed using Fisher’s exact test

Overall, 46.7% of the participants rejected the vaccine, 45.6% accepted, and 7.6% were vaccine hesitant. There was no difference between age groups, but females were less likely to accept vaccination than males (40.7%, 73.1% respectively, (*p* = 0.01)). There was no statistical difference between the three groups in regard of being a frontline worker, current practice model, having a chronic illness, or adherence to quarantine measures. The degree of perceived anxiety about COVID-19 was significantly different between the groups, as those who were not anxious about COVID-19 at all were more likely to accept the vaccine (81.8%), than those who were extremely anxious (34.9%) or somewhat anxious (46.2%) (Table 1).

Those who never postponed other recommended vaccines were more likely to accept the COVID-19 vaccine (60.6%) than those who postponed vaccines (17.3%). Moreover, believing that one is more likely to contract COVID-19 due to the nature of dental work was statistically significant between the three groups as those who disagreed were less likely to accept the vaccine (*p* < 0.01). Trusting the safety of vaccines (*p* < 0.01), and their effectiveness to prevent diseases (*p* = 0.031) were significantly different, while believing that recovering from a disease provided better immunity than vaccines was not significantly different between the groups. As for the COVID-19 vaccine in particular, those who were concerned about its ability to transmit the virus, its safety, its long-term side-effects, its potential to cause allergy, or to worsen their health condition, were significantly more likely to refuse the vaccine, while fertility concerns and concerns about fake vaccines were not significantly different. Those who were concerned about the teratogenicity of the vaccine had a higher refusal percentage, however, the difference was not statistically significant. A high percentage of the refusal group believed that the trials’ period was short (52.9%), expressed their willingness to receive the vaccine after it is administered to a larger proportion of the public (57.1%), and expressed their trust in the companies that developed the vaccines (64%), and these differences were statically significant. On the other hand, the majority of those who were accepting the vaccine believed that it would prevent them from contracting COVID-19 (78.1%) or prevent them from transmitting it to others (72%), compared to those who disagreed with these beliefs (23.3%, 18.2% respectively) (Table 2).

**Table 2:** Beliefs of the vaccine accepting, vaccine refusing, and vaccine hesitant dental teaching staff of Faculty of Dentistry, Ain Shams University, Egypt (*N* = 171)

		Are you willing to take COVID-19 vaccine?			Total	*P* value
		No	Not sure	Yes		
Have you ever postponed a vaccine recommended by the ministry of health/physician because you have doubts about it?	I don't remember	5 (50.0%)	2 (20.0%)	3 (30.0%)	10 (5.8)	< 0.01*
	No	41 (37.6%)	2 (1.8%)	66 (60.6%)	109 (63.7)	
	Yes	34 (65.4%)	9 (17.3%)	9 (17.3%)	52 (30.4)	
I am more likely to contract COVID-19 than other people because of the nature of my job.	Strongly agree/agree	55 (44.7%)	6 (4.9%)	62 (50.4%)	123 (71.9)	< 0.01*
	Neutral	14 (40.0%)	7 (20.0%)	14 (40.0%)	35 (20.5)	
	Strongly disagree/disagree	11 (84.6%)	0 (0.0%)	2 (15.4%)	13 (7.6)	
Even if I fall ill with another disease, I will not go to hospital because of risk of getting COVID-19 in the hospital.	Strongly agree/agree	26 (44.1%)	2 (3.4%)	31 (52.5%)	59 (34.5)	.484
	Neutral	31 (50.0%)	6 (9.7%)	25 (40.3%)	62 (36.3)	
	Strongly disagree/disagree	23 (46.0%)	5 (10.0%)	22 (44.0%)	50 (29.2)	
I believe I can protect myself against COVID-19 better than other people because I have a good medical background.	Strongly agree/agree	34 (47.2%)	4 (5.6%)	34 (47.2%)	72 (42.1)	.410
	Neutral	30 (52.6%)	6 (10.5%)	21 (36.8%)	57 (33.3)	
	Strongly disagree/disagree	16 (38.1%)	3 (7.1%)	23 (54.8%)	42 (24.6)	
I believe that the number of COVID-19 patients will increase in my country over the next month.	Strongly agree/agree	59 (50.9%)	6 (5.2%)	51 (44.0%)	116 (67.8)	.172
	Neutral	15 (42.9%)	5 (14.3%)	15 (42.9%)	35 (20.5)	
	Strongly disagree/disagree	6 (30.0%)	2 (10.0%)	12 (60.0%)	20 (11.7)	
I believe that community facilities such as educational institutions should be closed in the meantime.	Strongly agree/agree	55 (51.4%)	7 (6.5%)	45 (42.1%)	107 (62.6)	.079
	Neutral	14 (45.2%)	5 (16.1%)	12 (38.7%)	31 (18.1)	
	Strongly disagree/disagree	11 (33.3%)	1 (3.0%)	21 (63.6%)	33 (19.3)	
I believe that all international travel should be banned in the meantime.	Strongly agree/agree	53 (54.1%)	9 (9.2%)	36 (36.7%)	98 (57.3)	.076
	Neutral	15 (39.5%)	3 (7.9%)	20 (52.6%)	38 (22.2)	
	Strongly disagree/disagree	12 (34.3%)	1 (2.9%)	22 (62.9%)	35 (20.5)	
I expect this pandemic to get larger.	Strongly agree/agree	44 (55.0%)	6 (7.5%)	30 (37.5%)	80 (46.7)	.261
	Neutral	25 (42.4%)	5 (8.5%)	29 (49.2%)	59 (34.5)	
	Strongly disagree/disagree	11 (34.4%)	2 (6.3%)	19 (59.4%)	32 (18.7)	
Vaccines are effective at preventing diseases.	Strongly agree/agree	48 (42.9%)	5 (4.5%)	59 (52.7%)	112 (65.5)	.031*
	Neutral	22 (51.2%)	7 (16.3%)	14 (32.6%)	43 (25.1)	
	Strongly disagree/disagree	10 (62.5%)	1 (6.3%)	5 (31.3%)	16 (9.4)	
Diseases provide better immunity than vaccines do.	Strongly agree/agree	21 (53.8%)	2 (5.1%)	16 (41.0%)	39 (22.8)	.293
	Neutral	44 (50.0%)	8 (9.1%)	36 (40.9%)	88 (51.5)	
	Strongly disagree/disagree	15 (34.1%)	3 (6.8%)	26 (59.1%)	44 (25.7)	
I believe vaccines are safe.	Strongly agree/agree	14 (28.0%)	0 (0.0%)	36 (72.0%)	50 (29.2)	< 0.01*
	Neutral	41 (48.2%)	8 (9.4%)	36 (42.4%)	85 (49.7)	
	Strongly disagree/disagree	25 (69.4%)	5 (13.9%)	6 (16.7%)	36 (21.1)	
I’m concerned COVID-19 vaccine might transmit the virus to me.	Strongly agree/agree	27 (79.4%)	2 (5.9%)	5 (14.7%)	34 (19.9)	< 0.01*
	Neutral	21 (51.2%)	8 (19.5%)	12 (29.3%)	41 (24)	
	Strongly disagree/disagree	32 (33.3%)	3 (3.1%)	61 (63.5%)	96 (65.1)	
I’m concerned about the efficacy of COVID-19 vaccination.	Strongly agree/agree	61 (57.5%)	6 (5.7%)	39 (36.8%)	106 (62)	< 0.01*
	Neutral	15 (31.9%)	7 (14.9%)	25 (53.2%)	47 (27.5)	
	Strongly disagree/disagree	4 (22.2%)	0 (0.0%)	14 (77.8%)	18 (10.5)	
I’m concerned about the safety of COVID-19 vaccination.	Strongly agree/agree	69 (60.5%)	7 (6.1%)	38 (33.3%)	114 (66.7)	< 0.01*
	Neutral	7 (18.4%)	6 (15.8%)	25 (65.8%)	38 (22.2)	
	Strongly disagree/disagree	4 (21.1%)	0 (0.0%)	15 (78.9%)	19 (11.1)	
I’m concerned about the long-term side effects of COVID-19 vaccination.	Strongly agree/agree	68 (57.1%)	9 (7.6%)	42 (35.3%)	119 (69.6)	< 0.01*
	Neutral	10 (26.3%)	4 (10.5%)	24 (63.2%)	38 (22.2)	
	Strongly disagree/disagree	2 (14.3%)	0 (0.0%)	12 (85.7%)	14 (8.1)	
I’m concerned about faulty/fake COVID-19 vaccine.	Strongly agree/agree	48 (53.3%)	7 (7.8%)	35 (38.9%)	90 (52.6)	.302
	Neutral	19 (43.2%)	4 (9.1%)	21 (47.7%)	44 (25.7)	
	Strongly disagree/disagree	13 (35.1%)	2 (5.4%)	22 (59.5%)	37 (21.6)	
I’m concerned it might affect my fertility.	Strongly agree/agree	24 (64.9%)	1 (2.7%)	12 (32.4%)	37 (21.6)	.145
	Neutral	29 (42.6%)	7 (10.3%)	32 (47.1%)	68 (39.8)	
	Strongly disagree/disagree	27 (40.9%)	5 (7.6%)	34 (51.5%)	66 (38.6)	
I’m concerned it might be teratogenic.	Strongly agree/agree	37 (60.7%)	5 (8.2%)	19 (31.1%)	61 (35.7)	.052
	Neutral	26 (39.4%)	6 (9.1%)	34 (51.5%)	66 (38.6)	
	Strongly disagree/disagree	17 (38.6%)	2 (4.5%)	25 (56.8%)	44 (25.7)	
I’m concerned I might have allergy to COVID-19 vaccine.	Strongly agree/agree	27 (58.7%)	6 (13.0%)	13 (28.3%)	46 (26.9)	.039*
	Neutral	28 (43.8%)	5 (7.8%)	31 (48.4%)	64 (37.4)	
	Strongly disagree/disagree	25 (41.0%)	2 (3.3%)	34 (55.7%)	61 (35.7)	
I’m concerned it might make my current health condition worse.	Strongly agree/agree	48 (64.0%)	7 (9.3%)	20 (26.7%)	75 (43.9)	< 0.01*
	Neutral	20 (40.8%)	5 (10.2%)	24 (49.0%)	49 (28.7)	
	Strongly disagree/disagree	12 (25.5%)	1 (2.1%)	34 (72.3%)	47 (27.5)	
I believe the duration of clinical trials was short.	Strongly agree/agree	74 (52.9%)	9 (6.4%)	57 (40.7%)	140 (81.9)	< 0.01*
	Neutral	6 (30.0%)	4 (20.0%)	10 (50.0%)	20 (11.7)	
	Strongly disagree/disagree	0 (0.0%)	0 (0.0%)	11 (100.0%)	11 (6.4)	
I will only take the COVID-19 vaccine if the vaccine is taken by many in the public.	Strongly agree/agree	36 (57.1%)	6 (9.5%)	21 (33.3%)	63 (36.8)	.018*
	Neutral	25 (45.5%)	6 (10.9%)	24 (43.6%)	55 (32.1)	
	Strongly disagree/disagree	19 (35.8%)	1 (1.9%)	33 (62.3%)	53 (31)	
I don’t trust the pharmaceutical companies that developed the COVID-19 vaccine.	Strongly agree/agree	32 (64.0%)	1 (2.0%)	17 (34.0%)	50 (29.2)	< 0.01*
	Neutral	38 (50.0%)	10 (13.2%)	28 (36.8%)	76 (44.4)	
	Strongly disagree/disagree	10 (22.2%)	2 (4.4%)	33 (73.3%)	45 (26.3)	
I believe vaccination decreases my chances of getting COVID-19 or its complications.	Strongly agree/agree	14 (19.2%)	2 (2.7%)	57 (78.1%)	73 (42.7)	< 0.01*
	Neutral	45 (66.2%)	9 (13.2%)	14 (20.6%)	68 (39.8)	
	Strongly disagree/disagree	21 (70.0%)	2 (6.7%)	7 (23.3%)	30 (17.5)	
I would get the vaccine to prevent transmitting COVID-19 to relatives/friends.	Strongly agree/agree	19 (23.2%)	4 (4.9%)	59 (72.0%)	82 (48)	< 0.01*
	Neutral	37 (66.1%)	6 (10.7%)	13 (23.2%)	56 (32.7)	
	Strongly disagree/disagree	24 (72.7%)	3 (9.1%)	6 (18.2%)	33 (19.3)	

*Statistically significant differences. All data were analyzed using Fisher’s exact test

Female gender, not having a private practice, not intending to travel internationally, having anyone sick in the immediate social circle, and being more anxious about COVID-19 were significantly associated with unwillingness to receive the COVID-19 vaccine (indicated by “Not sure” or “No”), and thus these factors were included as predictors in the final model.

In the multivariate analysis, having a private job, and anxiety about COVID-19 were no longer significant predictors of willingness to receive the vaccine. However, unwillingness to receive the COVID-19 vaccine was still significantly higher among females (aOR = 0.34), and those who had someone in their immediate social network currently sick with COVID-19 (aOR = 0.37). While those who intended to travel within 2021 were 2.7 times more likely to accept the vaccine (Table 3).

**Table 3:** Predictors of willingness of dental teaching staff of the Faculty of Dentistry, Ain Shams University to receive COVID-19 vaccine (*N* = 171)

	Crude OR (95% CI)	***P*** value	aOR (95% CI)	***P*** value
Gender (female vs male^a^)	0.25 (0.1–0.64)	.004*	0.34 (0.12–0.98)	0.046*
Current practice (ASU^a^ vs ASU and private)	2.05 (1.11–3.78)	.022*	1.4 (0.69–2.86)	0.354
Intent to travel (no^a^ vs yes)	3.66 (1.51–8.88)	.004*	2.76 (1.05–7.26)	0.04*
Someone in the immediate social network contracted COVID-19 (no^a^ vs yes)	0.36 (0.18–0.72)	.004*	0.37 (0.18–0.77)	0.008*
Degree of anxiety about COVID-19	–	.037*	–	0.39
Degree of anxiety (somewhat vs not at all^a^)	0.19 (0.04–0.92)	.039*	0.37 (0.07–2.01)	0.25
Degree of anxiety (extremely vs not at all^a^)	0.12 ( 0.002–0.62)	.012*	0.29 (0.05–1.7)	0.17

For the multivariate regression model χ^2^ = 29, *P* value < 0.01, Nagelkerke *R*^2^ value = 20.9%.

*Statistically significant differences

^a^Reference category

## Discussion

In our study, 45.6% dental HCWs in the selected university were willing to receive the vaccine, while 46.7% were against vaccination, and 7.6% were vaccine hesitant. Our results showed higher acceptance rates compared to previous studies conducted on Egyptian HCWs. For example, a previous multi-national study [19] reported that only 24% of Egyptian HCWs were willing to receive COVID-19 vaccine. Another study by El-Sokkary et al. [11] reported an acceptance rate of 26% among Egyptian HCWs including dentists. However, a very small number of dentists participated in that study (22 dentists), and acceptance rate among them was only 6.2%, being very low compared to our study. Fares et al. [2] reported 21% acceptance rate among Egyptian physicians, nurses, pharmacists, physiotherapists, and dentists. However, only 13 dentists participated in that study, among which only two dentists accepted receiving COVID-19 vaccine, and eight of them were still hesitant. The differences in the acceptance rate between our findings and those of other studies might have occurred due to the difference in the time during which the study was conducted; our study was conducted after wider availability of vaccine and the presence of a higher number of vaccinated people, also more studies ensuring vaccine safety and efficacy were already conducted. Acceptance rate could have also varied due to differences in morbidity and mortality rates of COVID-19 across different times. The relatively higher acceptance rate seen in our study could also reflect the increasing awareness that dentists are at a higher risk of exposure to COVID-19 due to performing procedures in proximity to the oropharyngeal region [20].

Studies across different countries have also shown different vaccine acceptance rates among HCWs. For example, in France, vaccine acceptance rate reached 75% [21] among medical (physicians, pharmacists, nurses) and non-medical personnel. Acceptance rate also reached 70% among physicians, nurses, midwives, and medical technicians in Saudi Arabia [13]. While in Turkey, acceptance rate reached 68.6% [12] among physicians, nurses, midwives, and medical/nursing students. On the other hand, lower vaccine acceptance rates were noticed among HCWs in the USA, where acceptance rates were only 36% [14]. In Congo, as well, acceptance rate was only 27.7% among physicians, nurses and non-medical personnel [8].

In our study, social media was reported as the primary source of information about COVID-19 by most of our study sample. This finding agrees with a previous finding by Abu-Farha et al. [22] in Middle Eastern populations, and can be justified by the popularity and accessibility of social media networks, which was particularly more obvious during quarantine and lockdown. Although we cannot underestimate the power of social media, a major drawback of such means is the possibility of dissemination of rumors and false information which might negatively affect the public, causing vaccine hesitancy, delay, or rejection.

Several factors were identified to be significantly associated with vaccine acceptance. In our study, females were significantly less likely to accept the vaccine, with rejection rates of 51% compared to only 6% among males, and this difference remained statistically significant after adjusting for possible confounding factors. However, our study sample comprised a significantly higher number of females versus males (84.8% females versus 15.2% males) as the majority of the teaching staff in the Faculty of Dentistry, Ain Shams University, are females, this could have exaggerated the study results. Despite that, this finding was consistent with other studies that reported higher vaccine rejection rates and hesitancy among females [2, 11, 14, 15, 22]

Moreover, the perception of having a higher risk for contracting COVID-19 was significantly associated with more vaccine acceptance, which was in line with results obtained by Viswanath et al. [10]. In addition, responsibility and fear of transmitting the disease to relatives or friends was also a significant driver factor in receiving the vaccine. Surprisingly, perceived anxiety about COVID-19 was associated with less vaccine acceptance. However, we hypothesize that those individuals might be anxious about both, the disease and the vaccine, reflecting general anxiety.

Willingness to receive the vaccine was significantly higher among individuals who had intentions to travel internationally even after adjusting for other variables. This can be related to travel bans and restrictions in some countries that allow only fully vaccinated individuals to travel [23]. Lower willingness to receive the COVID-19 vaccine was unexpectedly noticed among individuals who had someone infected with COVID-19 in their close network. In our opinion, observing someone sick in one’s close network might have affected their perception of COVID-19’s threat, especially if the symptoms were not severe. Nevertheless, further studies are required to explore factors associated with changes in perceptions in those individuals.

Concerns about safety, efficacy, effectiveness, and long-term side effects of the vaccine were among the most statistically significant factors that hinder vaccine uptake by the study participants. Fear of allergies, worsening of the current health status, complications and disease transmission via the vaccine also led to a lack of confidence in the vaccine which adversely affected vaccine acceptance among DTS. Those findings were also reported by previous studies [10, 24] and could be hindering factors to potential herd immunity due to the existence of unvaccinated groups [10]. In addition, lack of confidence in pharmaceutical companies, the speed by which the vaccine was developed and the short duration for clinical trials were also statistically significant factors associated with vaccine hesitancy and rejection, this finding was also reported by Magadmi et al. [24]. Evidence of a lack of confidence in the vaccine also showed in our study when the majority of respondents reported that they might receive the vaccine only if it was taken by a large percentage of the public, which was also a statistically significant factor associated with vaccine acceptance. Also, individuals who had a previous experience with postponing a recommended vaccine were significantly more likely to reject COVID-19 vaccine than their counterparts.

### Limitations

Our study is not without limitations; first, our survey was conducted in a rapidly changing, dynamic environment, as individuals’ perceptions change on a daily basis according to the pandemic status, new information about the effectiveness and safety of the vaccines, and morbidity and mortality rates across the country. Second, the survey was conducted online to follow the restrictions on social distancing, therefore selection bias and accessibility issues should be considered. Third, the voluntary basis of participation in our study could have allowed self-selection bias by staff members who are particularly concerned about the pandemic. Finally, it is worth mentioning that this study is more skewed towards the female gender since the majority of the teaching staff in the faculty is females, which might limit the generalizability of this study, also, this study was conducted in one center, so the results could not be generalized on all dental HCWs in Egypt. Despite those limitations, this study was helpful in identifying vaccine hesitancy and perceptions towards COVID-19 vaccines among an essential group of HCWs. Future studies are required on a country-level to allow better exploration of potential factors of vaccine hesitancy among dental HCWs.

## Conclusion

This study reflects that a large number of Egyptian DTS are still not willing to receive the COVID-19 vaccine, which might hinder reaching herd immunity. Findings of our study can guide health authorities in Egypt to adopt strategies and interventions that correct misconceptions of individuals about COVID-19 vaccines and build their trust in the efficacy and safety of COVID-19 vaccines. Future research should also monitor the perceptions and attitudes of dental HCWs towards COVID-19 vaccines, as they are a reliable and a primary source of information to the public, and their perceptions can greatly influence the perceptions of the public in accepting or refusing the vaccine.

## Data Availability

The research data will be available upon a reasonable request to the corresponding author.

## Abbreviations

WHO: World health organization
HCW: Healthcare workers
DTS: Dental teaching staff
FD-ASU: Faculty of Dentistry, Ain Shams University
